# Supplementary material for: Reducing the risks of nuclear war – the role of health professionals
Source: Rev Saude Publica. 2023 Sep 14;57:1ed. doi: 10.11606/s1518-8787.20230570001ed (PMC10519673; doi:10.11606/s1518-8787.20230570001ed)
Supplement: Translation

# Translation: Reduzindo os riscos de uma guerra nuclear – o papel dos profissionais de saúde

**Authors:** Kamran Abbasi, Parveen Ali, Virginia Barbour, Kirsten Bibbins-Domingo, Marcel GM Olde Rikkert, Andy Haines, Ira Helfand, Richard Horton, Bob Mash, Arun Mitra, Carlos Monteiro, Elena N. Naumova, Eric J. Rubin, Tilman Ruff, Peush Sahni, James Tumwine, Paul Yonga, Chris Zielinski

**Affiliations:** I United Kingdom British Medical Journal, London, UK; II USA International Nursing Review, Sheffield UK; III Australia Medical Journal of Australia, Brisbane, Australia; IV USA JAMA, San Francisco, US; V The Netherlands Dutch Journal of Medicine, Nijmegen, The Netherlands; VI United Kingdom London School of Hygiene and Tropical Medicine, London, UK; VII USA Ex-Presidente, International Physicians for the Prevention of Nuclear War, Springfield, USA; VIII USA The Lancet, London, UK; IX South Africa African Journal of Primary Health Care e Family Medicine, Stellenbosch, South Africa; X USA International Physicians for the Prevention of Nuclear War, Ludhiana, India; XI Brazil Revista de Saúde Pública, São Paulo, Brazil; XII USA Journal of Public Health Policy, Boston, USA; XIII USA New England Journal of Medicine, Cambridge, USA; XIV USA International Physicians for the Prevention of Nuclear War, Melbourne, Australia; XV India National Medical Journal of India, New Delhi, India; XVI Uganda African Health Sciences, Kampala, Uganda; XVII Kenya East African Medical Journal, Nairobi, Kenya; XVIII United Kingdom University of Winchester, Winchester, UK

Em janeiro de 2023, o Conselho de Ciência e Segurança do Boletim dos Cientistas Atômicos avançou os ponteiros do Relógio do Juízo Final para 90 segundos antes da meia-noite, refletindo o risco crescente de uma guerra nuclear^1^. Em agosto de 2022, o secretário-geral da ONU, António Guterres, alertou que o mundo está agora em “um momento de risco nuclear inédito desde o auge da Guerra Fria”^2^. O risco tem sido marcado pelas crescentes tensões entre muitos estados munidos de armas nucleares^1 , 3^. Como editores de revistas médicas e de saúde em todo o mundo, pedimos aos profissionais de saúde que alertem o público e nossos líderes sobre esse grande risco para a saúde pública e os sistemas essenciais de suporte à vida do planeta – e exigimos ações para evitá-lo.

Os atuais esforços de controle de armas nucleares e de não-proliferação são inadequados para proteger a população mundial contra a ameaça de guerra nuclear, seja ela intencional, por engano ou por erro de cálculo. O Tratado de Não Proliferação de Armas Nucleares (TNP) compromete cada uma das 190 nações participantes “a prosseguir negociações de boa-fé sobre medidas eficazes relacionadas com a cessação da corrida ao armamento nuclear numa data precoce e com o desarmamento nuclear, e a um tratado para o desarmamento geral e completo sob controle internacional rigoroso e eficaz”^4^. O progresso tem sido decepcionantemente lento e a mais recente conferência de revisão do TNP em 2022 terminou sem uma declaração acordada^5^. Há muitos exemplos de quase desastres que expuseram os riscos de depender da dissuasão nuclear por um futuro indefinido^6^. A modernização dos arsenais nucleares pode aumentar os riscos: por exemplo, os mísseis hipersônicos diminuem o tempo disponível para distinguir entre um ataque e um alarme falso, aumentando a probabilidade de uma rápida escalada.

Qualquer uso de armas nucleares seria catastrófico para a humanidade. Mesmo uma guerra nuclear “limitada” envolvendo apenas 250 das 13 000 armas nucleares do mundo poderia matar 120 milhões de pessoas diretamente e causar perturbações climáticas globais, levando a uma fome nuclear, colocando 2 bilhões de pessoas em risco^7 , 8^. Uma guerra nuclear em larga escala entre os EUA e a Rússia poderia matar 200 milhões de pessoas ou mais no curto prazo, e potencialmente causar um “inverno nuclear” global que poderia matar de 5 a 6 bilhões de pessoas, ameaçando a sobrevivência da humanidade^7 , 8^. Uma vez que uma arma nuclear é detonada, a escalada para uma guerra nuclear total pode ocorrer rapidamente. A prevenção de qualquer uso de armas nucleares é, portanto, uma prioridade urgente de saúde pública e medidas fundamentais também devem ser tomadas para abordar a raiz do problema – abolir as armas nucleares.

A comunidade da saúde tem tido um papel crucial nos esforços para reduzir o risco de guerra nuclear e deve continuar a fazê-lo no futuro.^9^Na década de 1980, os esforços dos profissionais de saúde, liderados pela Médicos Internacionais para a Prevenção da Guerra Nuclear (IPPNW – *International Physicians for the Prevention of Nuclear War* ), ajudaram a acabar com a corrida armamentista da Guerra Fria, educando os formuladores de políticas e o público de ambos os lados da Cortina de Ferro sobre as consequências médicas da guerra nuclear. Isso foi reconhecido quando o Prêmio Nobel da Paz de 1985 foi concedido ao IPPNW (http://www.ippnw.org)^10^.

Em 2007, o IPPNW lançou a Campanha Internacional para a Abolição das Armas Nucleares, que se transformou em uma campanha global da sociedade civil com centenas de organizações parceiras. Um caminho para a abolição nuclear foi criado com a adoção do Tratado sobre a Proibição de Armas Nucleares em 2017, pelo qual a Campanha Internacional para Abolir as Armas Nucleares recebeu o Prêmio Nobel da Paz de 2017. Organizações médicas internacionais, incluindo o Comitê Internacional da Cruz Vermelha, a IPPNW, a Associação Médica Mundial, a Federação Mundial de Associações de Saúde Pública e o Conselho Internacional de Enfermeiros, tiveram papéis fundamentais no processo que antecedeu as negociações e nas próprias negociações, apresentando as evidências científicas sobre as consequências catastróficas para a saúde e o meio ambiente das armas nucleares e da guerra nuclear. Eles continuaram essa importante colaboração durante a Primeira Reunião dos Estados Partes do Tratado sobre a Proibição de Armas Nucleares, que atualmente conta com 92 signatários, incluindo 68 Estados membros^11^.

Apelamos agora às associações profissionais de saúde para que informem os seus membros do mundo inteiro sobre a ameaça à sobrevivência humana e se juntem à IPPNW para apoiar os esforços para reduzir os riscos de uma guerra nuclear a curto prazo, incluindo três medidas imediatas por parte dos estados com armas nucleares e seus aliados: primeiro, adotem uma política de não ao primeiro uso^12^; em segundo lugar, tirar suas armas nucleares do alerta de pronto lançamento e, em terceiro lugar, apelar a todos os estados envolvidos nos conflitos atuais a se comprometerem pública e inequivocamente que não usarão armas nucleares nesses conflitos. Pedimos-lhes ainda que trabalhem para um fim definitivo da ameaça nuclear, apoiando o início urgente de negociações entre os estados com armas nucleares para um acordo verificável e calendarizado para eliminar as suas armas nucleares em conformidade com os compromissos do TNP, abrindo caminho para que todas as nações integrem o Tratado sobre a Proibição de Armas Nucleares.

O risco é grande e crescente. Os estados com armas nucleares devem eliminar os seus arsenais nucleares antes de que eles nos eliminem. A comunidade da saúde desempenhou um papel decisivo durante a Guerra Fria e, mais recentemente, no desenvolvimento do Tratado de Proibição de Armas Nucleares. Temos de retomar este desafio como uma prioridade urgente, trabalhando com energia renovada para reduzir os riscos de uma guerra nuclear e eliminar as armas nucleares.
